# Epigenetic silencing of HIC1 promotes epithelial-mesenchymal transition and drives progression in esophageal squamous cell carcinoma

**DOI:** 10.18632/oncotarget.5832

**Published:** 2015-10-15

**Authors:** Pei Li, Xiang Liu, Zi-Ming Dong, Zhi-Qiang Ling

**Affiliations:** ^1^ Department of Pathophysiology, School of Basic Medical Sciences, Zhengzhou University, Zhengzhou, 450052, China; ^2^ Zhejiang Cancer Research Institute, Zhejiang Province Cancer Hospital, Zhejiang Cancer Center, Hangzhou 310022, China

**Keywords:** esophageal squamous cell carcinoma (ESCC), hypermethylated in cancer 1 (HIC1), epithelial-mesenchymal transition (EMT), promoter methylation, EphA2

## Abstract

Downregulation of the novel tumor suppressor gene HIC1 (hypermethylated in cancer 1) occurs frequently in various tumors where it causes tumor progression and metastasis. In this study, we investigated a role of HIC1 in esophageal squamous cell carcinoma (ESCC) and the underlying mechanisms. Downregulation of HIC1 occurred in approximately 70% of primary ESCCs at both mRNA and protein level where it was associated significantly with vascular invasion, advanced clinical stage, lymph node metastasis, and poor disease free survival (DFS). The promoter methylation analyses suggested that loss of HIC1 expression was mediated by epigenetic mechanisms. Functional studies established that ectopic re-expression of HIC1 in ESCC cells inhibited cell proliferation, clonogenicity, cell motility, tumor formation and epithelial-mesenchymal transition (EMT). Our results decipher the mechanism through which HIC1 deficiency induce ESCC cells to undergo EMT and promote tumor progression and metastasis through activation of EphA2 signaling pathway. Together, loss of the regulation of EphA2 pathway through HIC1 epigenetic silencing could be an important mechanism in the ESCC progression. We identify a novel pathway that linking HIC1 downregulation to EphA2-inducing EMT in ESCC cells and may shed light on the development of novel anti-tumor therapeutics.

## INTRODUCTION

Esophageal squamous cell carcinoma (ESCC) is one of the most common cancers in the world with extremely poor prognosis due to late presentation and rapid progression [1, 2]. Moreover, it is the eighth among most frequent cancers worldwide and the fifth most frequent cancers in developing countries [2–4]. In ESCC patients, invasion, metastasis and recurrence are the major causes of death [2, 5]. However, the mechanism of the development, invasion and metastasis of esophageal carcinoma is still not clear.

HIC1 (hypermethylated in cancer 1), as a new candidate tumor suppressor gene, located at 17p13.3 region telomeric to TP53 [6]. It has been proved that this region is frequently affected by genetic alterations such as deletion and epigenetic modifications like hypermethylation in human cancers, including the p53 tumor suppressor gene at 17p13.1 [6]. Accumulating dada showed HIC1 is epigenetically silenced in various types of common human cancers such as prostate cancers [7, 8], hepatocellular carcinoma [9, 10], pancreatic cancer [11], hyperparathyroid tumors [12], renal cell carcinoma [13], et al. HIC1 promoter methylation is associated with tumor aggressiveness and poor survival [14]. It has been shown that demethylation treatment restored HIC1 expression and impaired aggressiveness of head and neck squamous cell carcinoma [15]. These findings suggest that epigenetic HIC1 silencing predisposes tissues to tumorigenesis. However, the status and role of HIC1 by epigenetic modification in ESCC have never been analyzed in detail and thus still remain unsettled.

Epithelial-mesenchymal transition (EMT) is first recognized as a central feature of normal development and plays an important role in embryonic development [16]. In epithelial carcinomas, EMT is a process whereby cancer cells lose their epithelial properties to acquire a mesenchymal phenotype and become motile and invasive, which is closely associated with metastasis [17]. EMT has also been connected to induction of cancer stem cells, drug resistance, and immunosuppression [18–21], suggesting that EMT may underlie many biological processes related to tumor progression. RTK/Ras signaling has been found to contribute to EMT. The repression of E-cadherin has emerged as one important step driving EMT, and this stage is currently linked with some key molecules [22]. EphA2, also known as epithelial cell kinase (Eck), was found in adult human epithelial cells [23]. As a member of the family of receptor tyrosine kinases (RTK), EphA2 plays a critical role in embryonic patterning, neuronal targeting, vascular development, and tumor progression, especially in EMT, which it is an attractive target for cancer therapy [24–26]. The overexpression of EphA2 has been discovered in many cancers and is associated with primary tumor initiation, progression, angiogenesis and metastasis [23–27].

Here, we found that HIC1 promoter was hypermethylated in ESCCs and *in vitro* functions of HIC1 in ESCC are further investigated. We also identified that EphA2 is a potential downstream target gene of HIC1. These findings indicate that HIC1 by hypermethylation may play a critical role in facilitating ESCC progression.

## RESULTS

### HIC1 expression was regulated by promoter region hypermethylation in human esophageal squamous cell carcinoma cell lines

**Figure 1:**
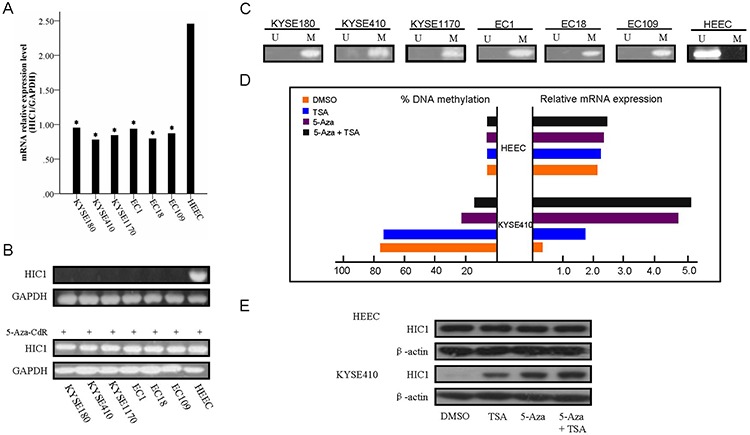
Expression of HIC1 is significantly reduced in ESCC cells The relative level of HIC1 transcript in ESCC cell lines in comparison with normal esophageal mucosa epithelial cell line examination is shown in **A.** HIC1 mRNA level was determined by RT-PCR and adjusted for GAPDH. * *P* < 0.05 vs HEEC. The mRNA level of HIC1 in ESCC cell lines as determined by 2.0% agarose gel electrophoresis, and 5-Aza-CdR treatments restored HIC1 gene expression in HIC1 silenced ESCC cell lines, were shown in **B.** Methylation status of HIC1 in ESCC cell lines is shown in **C** and **D.** 5-Aza-CdR or/and TSA treatments restored HIC1 gene expression in HIC1 silenced KYSE410 cells. Another esophageal mucosa epithelial cell line HEEC and KYSE410 were treated with 1.0 μmol/L 5-Aza-CdR for 72 hours and/or 100 nM TSA for 24 hours. The methylated levels were determined by real-time MSP. We performed real-time RT-PCR analysis in triplicate for each cDNA sample and used median values in three experiments. The relative HIC1 mRNA expression was normolized to the GAPDH of the same samples using the formula 2^−ΔΔCT^. The results were multiplied by 100 for a better visualization. The percentage of HIC1 DNA methylation is shown on the left side; whereas the relative mRNA expression of HIC1 is shown on the right side. **E.** Expression of HIC1 at the protein level following 5-Aza-CdR and TSA treatments. Western blot analysis of KYSE410 and HEEC cells following treatment with DMSO (control), 5-Aza-CdR, or 5-Aza-CdR/TSA for 72 hours demonstrate up-regulation of the HIC1 proteins in treated cells as compared to control (DMSO). β-actin is shown as a loading control.

The expression and the methylation status of HIC1 were detected by real-time RT-PCR and MSP in human esophageal squamous cell carcinoma cell lines. Downregulation of HIC1 was detected in all six ESCC cell lines (KYSE180, KYSE410, KYSE1170, EC1, EC18 and EC109), while the normal expression of HIC1 was detected in human normal esophageal epithelial cell line HEEC (Figure 1A and 1B). Complete methylation was detected in all six ESCC cell lines cells (Figure 1C). Restoration of HIC1 expression was revealed by 5-Aza-CdR treatment in six ESCC lines (Figure 1B), accompanied by demethylation of HIC1 promoter, while no change in HEEC cells (Figure 1D and 1E), indicating that HIC1 is transcriptionally silenced in these cells by DNA hypermethylation. Interestingly, TSA treatment alone was effective in restoring HIC1 expression in these ESCC lines cells without significant change of HIC1 methylation level, suggesting that histone modifications may also be involved in regulating HIC1 expression, which was very similar to my recent study [28]. However, administration of TSA following 5-Aza-CdR had an additive effect in restoring gene expression with a further decrease in the methylation level of HIC1 (Figure 1D). These results are in agreement with the previous study that suggested that TSA can have a demethylation effect in a gene-specific manner [29]. The western blot analysis using KYSE410 and HEEC cells, as a model, confirmed the upregulation of HIC1 proteins following the 5-Aza-CdR and 5-Aza-CdR/TSA treatments in KYSE410 cells, while no change in HEEC cells (Figure 1E).

### HIC1 was frequently methylated and the expression was reduced in human primary ESCC tissues

**Figure 2:**
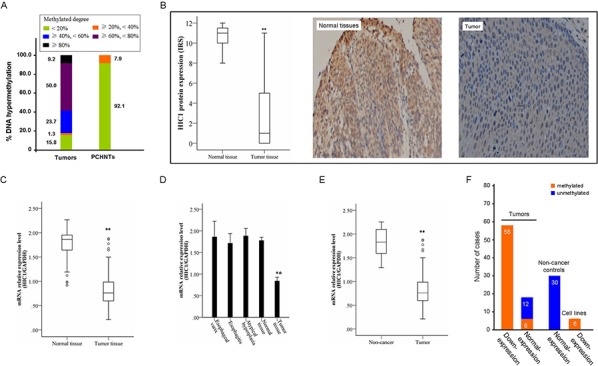
Representative results of HIC1 methylation and expression in primary ESCC **A.** Summary of HIC1 methylation in 76 primary ESCC tissues and paired para-cancerous histological normal tissues (PCHNTs) from the same patients. Different color block shows the methylated degree of HIC1 gene. Number representatives the frequency of HIC1 methylation with different degree in 76 cases with primary ESCC. **B.** The protein level of HIC1 in 76 primary ESCC tissues as determined by IHC. IHC score of 0–4 is considered to be no to low expression and 5–12 considered to be normal to high expression. The level of Ndrg2 expression in PCHNTs was higher than that in tumor tissues (** *P* < 0.01). Representative of HIC1 expression in a pair of ESCC and adjacent normal tissue detected by immunostaining with anti-HIC1 antibody (Right). The slide was counterstained with hematoxylin. Original magnification × 200. **C.** HIC1 mRNA level was determined by RT-PCR and adjusted for GAPDH. ** *P* < 0.01 vs PCHNTs. Increased and reduced expressions were defined as the median value of relative gene expression level > 2.0 and < 0.5, respectively. **D.** Comparison of expression level of HIC1 mRNA between esophageal cancer tissues and esophageal epithelium atypical hyperplasia, esophagitis, and esophageal varices. Normal tissues: paired PCHNTs. (** *P* < 0.01 vs each normal tissue samples). **E.** Comparison of expression level of HIC1 mRNA between esophageal cancer tissue and non-cancer tissue samples; non-cancer tissue samples: the mean of HIC mRNA level in 15 cases of esophageal epithelium atypical hyperplasia, 10 cases of esophagitis and 5 cases of esophageal varices. **F.** The association of HIC1 methylation and its mRNA expression was analyzed. The ESCC cell lines vs HEEC ratio or primary ESCC tissues vs paired PCHNTs or non-cancer controls vs HEEC < 0.5 is considered as low expression and > 2 to be considered as high expression. Number: the numbers of cases.

To analyze the methylation status of HIC1 in 76 human primary ESCCs were detected by real-time MSP. As shown in Figure 2A, 84.2% (64/76) of ESCCs was methylated, while only 7.9% (6/76) of its corresponding para-cancerous histological normal tissues (PCHNTs) was methylated, showing partial methylation. The percentage of HIC1 non-methylation, partial methylation, and complete methylation in 76 ESCC tissues was 15.8% (12/76), 75% (57/76) and 9.2% (7/76), respectively. The frequency of HIC1 methylation in ESCC tissues was significantly higher as compared with that in paired PCHNTs (*P* = 0.000). HIC1 expression both at mRNA and protein level was evaluated by real-time RT-PCR and immunohistochemistry analysis in 76 cases of available matched ESCC and adjacent normal tissues, respectively. The expression of HIC1 both at mRNA and protein level was reduced significantly in primary ESCC compared with paired adjacent normal tissues (both *P* = 0.000, Figure 2B and 2C). Meanwhile, we examined mRNA expression of HIC1 in esophageal epithelium atypical hyperplasia (*n* = 15), esophagitis (*n* = 10), and esophageal varices specimens (*n* = 5). As shown in Figure 2D, normal expression of HIC1 mRNA was detected in these non-cancer controls, and there was no significant difference of HIC1 expression in adjacent normal tissues as compared to each non-cancer controls (all *P* > 0.05). However, the HIC1 expression was significantly reduced in ESCC samples as compared with that in 3 esophageal benign lesion specimens (all *P* = 0.000) (Figure 2D) or in all non-cancer controls (*P* = 0.000) (Figure 2E). Correspondingly, the frequency of HIC1 methylation in all non-cancer controls was very low, even lower than that in adjacent normal tissues. Reduced expression was associated with promoter region hypermethylation (Figure 2F). These results suggest that HIC1 expression is possibly regulated by promoter region methylation in ESCC cells.

### HIC1 promoter hypermethylation is associated with its transcriptional silencing in ESCC cells

**Figure 3:**
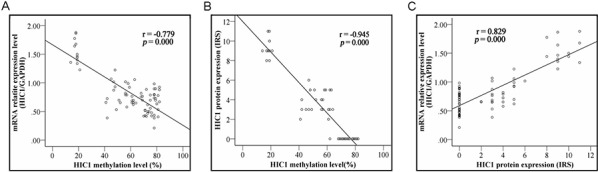
Correlation of HIC1 methylation with its expression Correlation of HIC1 methylation with HIC1 mRNA level determined by real-time RT-PCR analysis and HIC1 protein expression determined by immunohistochemical analysis in 76 ESCC tissues. HIC1 methylation scores inversely correlated with HIC1 gene expression both at mRNA and protein levels (both *P* = 0.000).

To examine the relationships between HIC1 methylation and HIC1 expression, we compared the HIC1 methylation level with HIC1 mRNA level detected by real-time RT-PCR and protein level determined by immunohistochemical analysis by the Spearman correlation analysis. HIC1 methylation level was significantly associated with HIC1 mRNA level (*r* = −0.779, *P* = 0.000) (Figure 3A) and HIC1 protein level (*r* = −945, *P* = 0.000) (Figure 3B). These results strongly indicted that HIC1 expression was regulated by HIC1 promoter methylation. More important, HIC1 protein expression determined by immunohistochemical analysis in 76 ESCC tissues was closely correlated with HIC1 mRNA level [lg(T/N)] (determined by RT-PCR) (*r* = 0.829, *P* = 0.000) (Figure 3C). Downregulation of HIC1 at mRNA and protein level by methylation was associated with late stage, vascular invasion and lymph node metastasis, respectively (Table 1, all *P* < 0.05).

**Table 1:** Correlation between HIC1 mRNA, HIC1 protein, HIC1 methylation and clinicopathologic parameters, respectively

Clinicopathological factors		Number of cases	HIC1 mRNA down-expression (%)	χ^2^	P*	HIC1 protein down-expression (%)	χ^2^	P*	HIC1 methylation (%)	χ^2^	P*
Cases											
	Tumor	76	58 (76.32)	97.121	**0.000**	55 (72.37)	86.186	**0.000**	64 (84.21)	89.082	**0.000**
	PCHNT	76	0 (0.00)			0 (0.00)			6 (7.89)		
Age at surgery											
	>60	37	30 (81.08)	0.906	0.341	28 (75.68)	394	0.530	33 (89.19)	1.344	0.246
	≤60	39	28 (71.79)			27 (69.23)			31 (79.49)		
Gender											
	Male	61	49 (80.33)	1.743	0.187	47 (77.05)	2.304	0.129	54 (88.52)	2.838	0.092
	Female	15	9 (60.00)			14 (93.33)			10 (66.67)		
Localization											
	Upper-Mid	46	33 (71.74)	1.35	0.245	31 (67.39)	1.444	0.230	36 (78.26)	2.072	0.150
	Lower	30	25 (83.33)			24 (80.00)			28 (93.33)		
Diameter											
	<5cm	36	26 (72.22)	0.634	0.426	26 (72.22)	0.001	0.978	29 (80.56)	0.687	0.407
	≥5cm	40	32 (80.00)			29 (72.50)			35 (87.50)		
Histological differentiation											
	High-Mid	61	46 (75.41)	0.001	0.972	43 (70.49)	0.173	0.678	51 (83.61)	0	1.000
	Low	15	12 (80.00)			12 (80.00)			13 (86.67)		
Vascular invasion											
	Negative	28	16 (57.14)	9.016	**0.003**	14 (50.00)	11.093	**0.001**	20 (71.43)	4.032	**0.045**
	Positive	48	42 (87.50)			41 (85.42)			44 (91.67)		
Tumor status											
	T1/T2	16	8 (50.00)	6.03	**0.014**	8 (50.00)	3.753	0.053	11 (68.75)	2.319	0.128
	T3/T4	60	50 (83.00)			47 (78.33)			53 (88.33)		
Lymph node status											
	No	28	16 (57.14)	9.016	**0.003**	13 (46.43)	14.918	**0.000**	20 (71.43)	4.032	**0.045**
	Yes	48	42 (87.50)			42 (87.50)			44 (91.67)		
Stage											
	I/II	35	20 (57.14)	13.195	**0.000**	17 (48.57)	18.373	**0.000**	26 (74.29)	4.806	**0.028**
	III/IV	41	38 (92.68)			38 (92.68)			38 (92.68)		
Smoking											
	No	24	18 (75.00)	0.034	0.855	16 (66.67)	0.57	0.450	19 (79.17)	0.231	0.631
	Yes	52	40 (76.92)			39 (75.00)			45 (86.54)		
Drinking											
	No	32	23 (71.88)	0.603	0.437	20 (62.50)	2.692	0.101	27 (84.38)	0.001	0.973
	Yes	44	35 (79.55)			35 (79.55)			37 (84.09)		
Family history of cancer											
	No	60	47 (78.33)	0.221	0.638	45 (75.00)	0.46	0.497	53 (83.33)	2.319	0.128
	Yes	16	11 (68.75)			10 (62.50)			11 (68.75)		

Down: T/N < 0.5 in mRNA, IHC score ≤4.

M: methylated degree ≥20%.

*P*: Pearson Chi-Square Tests.

### Effect of HIC1 downexpression on the prognosis of ESCC patients

The significant correlation of HIC1 downexpression with many clinico-pathological parameters (Table 1) suggested that it may associate with the prognosis of ESCC patients. Until the due date of Follow-up, 5 patients had no information. The loss ratio of follow-up was 6.6%. 54.5% (30/55) patients with HIC1 downexpression at protein level in ESCC tissues went rapid disease progression or died. The Median overall survival (OS) was only 7.4 months. In contrast, in the 21 patients with normal or upregulation of HIC1 protein in their ESCC tissues, 42.9% (9/21) patients were deteriorating, and the Median OS was 13.7 months. Kaplan-Meier analysis demonstrated that no significant correlation was found between patients’ OS and HIC1 protein downexpression, or HIC1 mRNA downexpression, or HIC1 methylation.

**Figure 4:**
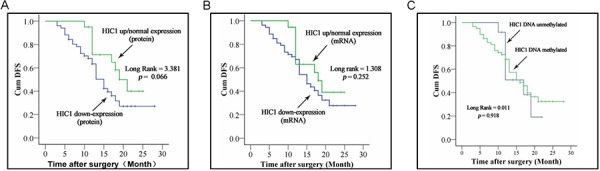
Correlation of HIC1 expression level and its methylated status with survival of ESCC patients Kaplan-Meier curves of survival durations in ESCC patients treated with primary surgical resection according to the expression level of HIC1 and its methylated status. Note that the survival duration was worse only in patients with HIC1 protein downregulation than those with of HIC1 normal or high expression.

Kaplan-Meier analysis demonstrated that patients with HIC1 protein downexpression in their ESCC tissues exhibited a relatively worse disease free survival (DFS) than that its normal or upregulation (*P* = 0.066) (Figure 4A). However, Kaplan-Meier analysis proved that patients with HIC1 mRNA downexpression had not significantly lower DFS than that in the patients with HIC1 mRNA normal or upexpression (*P* = 0.252) (Figure 4B). Consistent with the results from HIC1 mRNA, HIC1 promoter methylation in tumor tissues was not found to be an unfavorable predictor for the ESCC patients (*P* = 0.918) (Figure 4C).

Cox regression analysis revealed that HIC1 protein downexpression in tumor tissues was an independent factor on patients’ DFS: patients with HIC1 protein downexpression had worse prognosis (*P* = 0.024; Hazard ratio, 0.296; 95% CI, 0.102~0.855). Furthermore, it can be affected by such factors as TNM stages, gender, tumor localization and so on (Table 2).

**Table 2:** Univariate and multivariate analysis of survival in 76 ESCC patients according to clinicopathologic factors and HIC1 protein expression

Clinicopathologic factor		Number of case	Survival (mo)	DFS				
				Univariate analysis		Multivariate analysis^§^		
				χ^2^	*P*-values	HR	95% CI	*P*-values
Age at surgery											
	> 60	37	17	0.262	0.609	0.584	0.266–1.282	0.180
	≤ 60	39	15					
Gender								
	Male	61	15	0.079	0.779	10.447	1.648–66.21	0.013
	Female	15	18					
Localization								
	Upper-Mid	46	17	0.003	0.954	2.577	1.072–6.197	0.034
	Lower	30	15					
Diameter								
	<5cm	36	15	0.171	0.680	0.867	0.403–1.866	0.715
	≥5cm	40	19					
Histological differentiation								
	High-Mid	61	16	0.005	0.942	0.849	0.369–1.953	0.701
	Low	15	17					
Vascular invasion								
	Negative	28	13	0.304	0.582	0.473	0.162–1.375	0.169
	Positive	48	16					
Tumor status								
	T1/T2	16	17	0.028	0.866	0.199	0.049–0.8	0.023
	T3/T4	60	17					
Lymph node status								
	No	28	18	0.386	0.535	0.102	0.016–0.657	0.016
	Yes	48	15					
Stage								
	I/II	35	19	2.274	0.132	26.261	2.936–234.913	0.003
	III/IV	41	13					
Smoking								
	No	24	18	0.960	0.327	5.565	1.333–23.234	0.019
	Yes	52	15					
Drinking								
	No	32	18	3.623	0.057	1.933	0.732–5.103	0.183
	Yes	44	13					
Family history of cancer								
	No	60	17	0.445	0.505	2.268	0.882–5.834	0.089
	Yes	16	13					
HIC1 protein								
	Down	55	15	3.381	0.066	0.296	0.102–0.855	0.024
	Up/Normal	21	19					

### HIC1 expression level was associated with EMT features of ESCC

**Figure 5:**
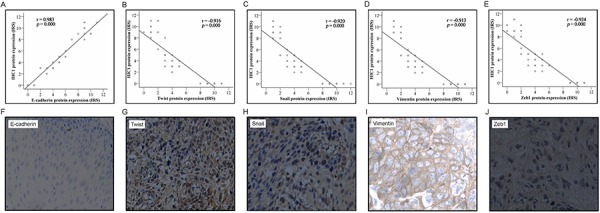
Correlation of HIC1 expression level with epithelial-mesenchymal transition (EMT) markers The expression level of HIC1 and EMT markers including E-cadherin, Twist, Snail, vimentin, and Zeb1 was determined by IHC analysis. The IHC score of HIC1 was plotted with the IHC score of EMT markers. Note that HIC1 expression level was positively correlated with E-cadherin but negatively correlated with Twist, Snail, Vimentin, and Zeb1 **A~E.** Representative immunohistochemical results of E-cadherin, Twist, Snail, Vimentin, and Zeb1 in ESCCs with HIC1 downexpression **F~J.**

Numerous studies have pinpointed that EMT is a key determinant for metastasis of human cancers [30]. As we found that HIC1 downexpression was strongly associated with increased metastasis of ESCC, we analyzed EMT markers including E-cadherin, Twist, Vimentin, Snail and Zeb1 with the ESCC samples. IHC scores revealed that HIC1 downexpression was strongly correlated with a decrease in the expression of E-cadherin (r = 0.983, *P* = 0.000) and an increases in the expression of Twist (*r* = − 0.916, *P* = 0.000), Snail (γ = − 0.920, *P* = 0.000), Vimentin (*r* = − 0.913, *P* = 0.000), and Zeb1 (r = − 0.924, *P* = 0.000) (Figure 5). These data, therefore, suggested that low expression of HIC1 was associated with an increase in EMT features, likely contributing to the observed aggravation of tumor metastasis in ESCC with reduced expression of HIC1.

### Suppression of cell growth and migration of ESCC cells by HIC1

**Figure 6:**
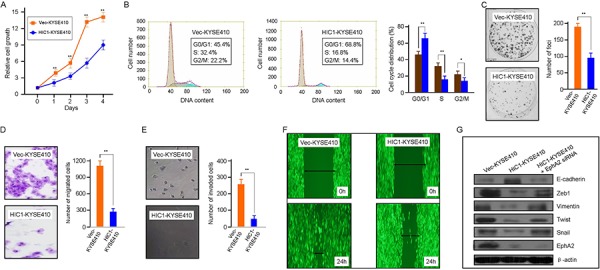
Tumor-suppressive function of HIC1 in ESCC cells **A.** HIC1 inhibits cell growth in HIC1-KYSE410 cells. Vec-KYSE410 cells expressing empty vector (control) or HIC1-KYSE410 cells were used to determined cell growth rate by MTT assay at indicated time points. The data are shown as mean ±SD, and bars, SD, ** for *P* < 0.01 by Student's *t*-test. **B.** HIC1 induces G0/G1 cell-cycle arrest. Representative and summary of DNA content detected by flow cytometry showed that the percentage of cells in S phase and G2/M was much lower in HIC1-KYSE410 cells than that in control cells (Vec-KYSE410 cells). Values are the mean ± SD of 3 independent experiments. **: *P* < 0.01; *: *P* < 0.05. **C.** Effect of HIC1 overexpression on colony formation in HIC1-KYSE410 cells. HIC1-KYSE410 cells and Vec-KYSE410 cells were seeded in to 6-well with 400 cells per well and then cultured for 5 days, respectively, followed by crystal violet staining and colony counting. Quantitative analyses of foci numbers (right). Columns, mean of at least 3 independent experiments; bars, SD. **: *P* < 0.01 versus controls by using the Student's *t* test. **D.** HIC1 upregulation reduced HIC1-KYSE410 cells migration. HIC1-KYSE410 cells and Vec-KYSE410 cells were seeded into 24-well transwell chambers and the cells on lower chamber were fixed in 24 h and stained with crystal violet, respectively. Number of migrated tumor cells was quantified in the right panel. Columns, mean of triplicate experiments. **, *P* < 0.01. **E.** Representative images showed the HIC1-KYSE410 cells and Vec-KYSE410 cells that invaded through the matrigel. Number of invaded tumor cells was quantified in the right panel. Columns, mean of triplicate experiments. **, *P* < 0.01. **F.** A scratched-wound healing assay was carried out with KYSE410 cells transfected with control plasmid (Vec-KYSE410) or HIC1 overexpressing plasmid (HIC1-KYSE410), followed by photography before and at 24 h after the scratch. **G.** Alteration of EMT markers and EphA2 by HIC1 overexpression. KYSE410 cells transfected with control plasmid (Vec-KYSE410) or HIC1 overexpressing plasmid HIC1-KYSE410) were cultured in complete medium for 2 days before immunoblotting with the antibodies as indicated.

We next investigated whether HIC1 had a direct inhibition on the growth and migration of ESCC cells. KYSE410 cell line was established from the poorly differentiated invasive esophageal squamous cell carcinoma resected from the cervical esophagus of a 51-year-old Japanese man prior to treatment (tumor invasion into the adventitia was obvious) (http://www.dsmz.de/mutz/mutzhome.htm). As analyzed by MTT assay, the cell proliferation rate of KYSE410 cells was significantly inhibited by HIC1 overexpression (Figure 6A). In the HIC1-KYSE410 cells, the mRNA level of HIC1 was about 11-fold of the control. HIC1 induces G0/G1 cell-cycle arrest. Representative and summary of DNA content detected by flow cytometry showed that the percentage of cells in S phase and G2/M phase were much lower in HIC1-KYSE410 cells than that in Vec-KYSE410 cells, while the percentage of cells in G0/G1 phase were much higher in HIC1-KYSE410 cells than that in controls (Figure 6B). Consistently, colony formation was also reduced by HIC1 overexpression (Figure 6C). In addition, the migration and invasion of HIC1-KYSE410 cells as analyzed by transwell assay and invasion assays were significantly inhibited by HIC1 overexpression (Figure 6D and 6E). Wound healing assay showed that cell motility was inhibited by HIC1 (Figure 6F). In agreement with previous findings that HIC1 is able to inhibit the receptor tyrosine kinase EphA2 [31], the activations of Eph pathway as judged by EphA2 were markedly inhibited by HIC1 overexpression (Figure 6G). Furthermore, the EMT features was also inhibited in these HIC1-KYSE410 cells, shown as an increase in epithelial markers E-cadherin together with a reduction in mesenchymal markers Zeb1, Vimentin, Twist and Snail upon HIC1 overexpression in HIC1-KYSE410 cells (Figure 6G).

**Figure 7:**
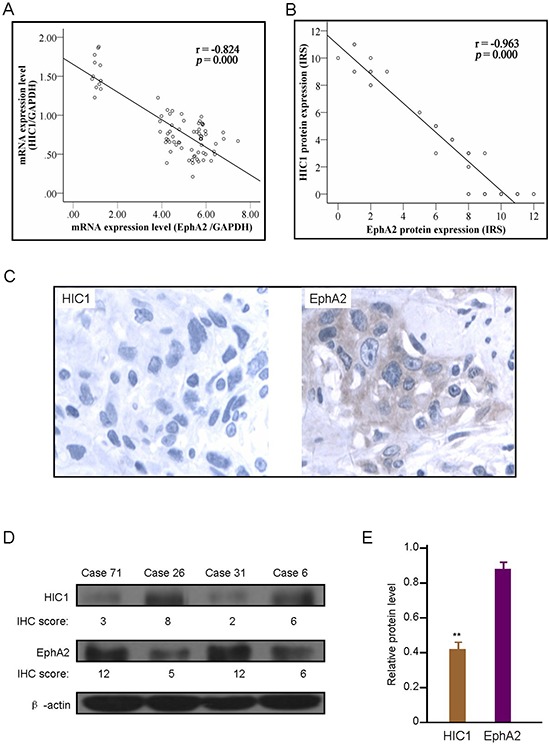
Correlation of HIC1 expression level with EphA2 expression **A.** Correlation of HIC1 mRNA expression with EphA2 mRNA level determined by real-time RT-PCR analysis in 76 ESCC tissues. **B.** Correlation of HIC1 protein expression with EphA2 protein expression determined by immunohistochemical analysis in 76 ESCC tissues. **C.** Representative immunohistochemical (IHC) images of HIC1 and EphA2 expression in same case. × 200 for all images. **D.** Representative western blotting images of HIC1 and EphA2 expression, and in comparison with IHC results in 4 randomly selected primary ESCC tissue samples. β-actin was internal control. **E.** Western blot analysis of HIC1 and EphA2 protein in same ESCC tissues showed that significant negative correlation was existed between HIC1 and EphA2 protein (** *P* < 0.01).

To examine the relationship between HIC1 and EphA2 expression, we compared their mRNA level by real-time RT-PCR or protein determined by IHC using the Spearman correlation analysis. HIC1 mRNA negatively related to EphA2 mRNA level detected by real-time RT-PCR (*r* = −0.824, *P* = 0.000) (Figure 7A). And HIC1 protein expression was closely negatively correlated with EphA2 protein level determined by IHC analysis (*r* = −0.963, *P* = 0.000) (Figure 7B). The negative correlation of HIC1 with EphA2 protein expression by IHC analysis were found in the same samples (Figure 7C). The western blot analyses was carried out to confirm the relationship between HIC1 and EphA2 protein expression in 20 randomly selected primary ESCC tissue samples. Some representative western blot results are shown in Figure 7D.

Collectively, these data indicated that HIC1 has a direct suppression on cell proliferation, migration, invasion and EMT of ESCC cells through the inhibition of EphA2 signaling pathway, consistent with our clinical findings that HIC1 expression level was negatively correlated with the malignancy and metastasis of ESCC cells.

## DISCUSSION

HIC1, as a new candidate tumor suppressor gene, located at 17p13.3 region telomeric to TP53 [6]. HIC1 downregulation is frequently found in a wide variety of tumors [6–13]. This inactivation of HIC1 might impel cancer cells to alter survival and signaling pathways or lineage-specific transcription factors during the early stages of tumorigenesis [32]. Here, we investigated the biological function and clinical significance of HIC1 in ESCC progression. We found that HIC1 was frequently downregulated in ESCC tissues and provided evidence suggesting that this observation can have significant implications in the negative regulation of ESCC progression. Our data suggested that HIC1 might be a novel candidate suppressor of tumor progression in ESCC. First, HIC1 downregulation both at mRNA and protein level was frequently observed in 76 primary ESCC tumors and six ESCC cell lines. Results showed that HIC1 expression was significantly reduced in the majority of ESCC tissues compared with adjacent non-tumor tissues, which was similar to other tumors [7–14]. HIC1 protein expression detected by immunohistochemical analysis in ESCC tissues indicated that HIC1 downregulation was closely correlated with vascular invasion, lymph node metastasis and clinical stage, respectively (all *P* < 0.05) (Table 1). Although hazard ratio is not obvious, we also found in the present study that HIC1 downexpression is one of the affecting factors on tumor progression by multivariate analysis (*P* = 0.024). Together, these data indicated that HIC1 might have a clinical significance as a marker associated with negative regulation of tumor progression in ESCC.

Emerging evidence suggests that HIC1 is frequently hypermethylated as a result of silence or low level in a variety of solid tumors [7–14]. Additionally, several posttranslational regulatory mechanisms have been described for affecting HIC1 function [32, 33]. Here, we showed that the CpG sites in the HIC1 promoter were mostly hypermethylated in primary ESCC tumors and cell lines, whereas those in normal esophageal mucosal tissues remained non-methylated. We concluded that the inactivation mediated by promoter methylation might be the major cause of the frequent HIC1 downregulation in ESCC cells. Using two *in vitro* cells model of KYSE410 and HEEC, we confirmed that DNA methylation was the mechanism underlying HIC1 gene silencing. 5-Aza-dC treatment significantly restored HIC1 expression in HIC1 silenced ESCC cell line KYSE410. However, HIC1 expression in normal esophageal epithelium line HEEC cells was not affected by 5-Aza-dC treatment. These data clearly proved that to a certain extent the frequent HIC1 downexpression in ESCC cells was regulated by HIC1 promoter hypermethylation. Results showed that both HIC1 hypermethylation and HIC1 downregulation both at mRNA and protein level were significantly correlated with vascular invasion, lymph node metastasis and clinical stage, respectively (all *P* < 0.05), suggesting that frequent dysfunction of HIC1 through its promoter methylation might play crucial roles in malignant progression of human esophageal cancer and might have a important impact on the metastasis and poor survival of ESCC patients. It was reported that the loss of suppressive function of HIC1 by promoter hypermethylation was responsible for prostate cancer progression and invasion [7]. Recently, Eggers H, et al pinpointed that HIC1 hypermethylation was associated with reduced recurrence-free survival in renal cell carcinoma (RCC), suggesting that HIC1 could be seen as a possible marker to improve individualized therapy and risk stratification [13].

The metastasis of ESCC was significantly correlated with HIC1 downexpression, which was one of the most important discoveries ever made by this study. Consistent with such observation, EMT features were well correlated with the expression level of HIC1, suggesting that HIC1 downexpression may enhance tumor metastasis via promotion of EMT. This hypothesis was further supported by our findings that the invasion and migration of ESCC cells was negatively regulated by HIC1 and EMT features were inhibited by HIC1 overexpression in HIC1-KYSE410 cells. Some studies have shown that Eph-mediated signaling pathways was implicated in the initiation of EMT [34], and HIC1 was involved the transcriptional regulation of the tyrosine kinase receptor EphA2. The abnormal regulation of Eph pathway via HIC1 epigenetic silencing could be an important mechanism in the pathogenesis of epithelial cancers [31]. Therefore, HIC1 could inhibit EMT through inhibition of EphA2-mediated pathways, supported by our findings that EphA2 upregulation was negatively activated by HIC1 in ESCC cells. The observed frequent HIC1 downregulation in ESCC cells, therefore, could be considered as another mechanism contributing to activation of EphA2 signaling commonly seen in ESCC cells. It is therefore imperative in the future to fully uncover the molecular mechanisms underlying the regulatory roles of HIC1 on the progression and metastasis of ESCC. Nevertheless, our findings that HIC1 downexpression was associated with the progression and prognosis of ESCC have heralded this molecule to be a promising therapeutic target for intervention of ESCC in the future.

## MATERIALS AND METHODS

### Cell culture and tumor specimens

Three Chinese ESCC cell lines (EC1, EC18 and EC109) were from our laboratory (Department of Pathophysiology, School of Basic Medical Sciences, Zhengzhou University). Three Japanese ESCC cell lines (KYSE180, KYSE410, KYSE1170) were kindly provided by Dr. Takeshi Tatsuta (Department of Surgery, Shiga University of Medical Science, Otsu 520–2192, Japan). The human esophageal epithelial cell line HEEC was obtained from the Resource Centre for Biological Material, Shanghai Institute for Biological Sciences, Chinese Academy of Sciences. These cell lines were used in this study, and were maintained in a 1:1 mixture of RPMI 1640 (Invitrogen, Carlsbad, CA) and Ham's F12 (Nissui Pharmaceutical, Tokyo, Japan) containing 10% fetal bovine serum (FBS, Gibco BRL Life technologies Inc., Rockville, MD, USA) in humidified 5% CO_2_-air at 37°C, respectively. To avoid possible effects on gene expression, antibiotic and antimycotic drugs were not used in the cell culture. 5-aza-2′-deoxycytidine (5-Aza-CdR) and trichostatin A (TSA) (Sigma-Aldrich, USA) were dissolved in dimethyl sulfoxide (DMSO). Cultured cells were seeded at a density of 5 × 10^5^ cells per flask. The cells were in logarithmic phase and the number of viable cells was 95% to 100% before the addition of 5-Aza-CdR to the culture medium at a final concentration of 1.0 μmol/L. The medium was changed daily and the drug concentration was maintained. The cells were collected after 72 h of drug treatment. Cells were exposed to TSA at a final concentration of 20 ng/ml for 24 hours. Cells in the untreated group were cultured in normal complete culture medium for 72 h.

Seventy-six ESCC specimens and their correspondent non-neoplastic tissues were acquired from surgeries in Zhejiang Province Cancer Hospital between February and July in 2010. The correspondent non-neoplastic tissues were cut from areas 5 cm outside the edge region of tumor and confirmed by microscopy as normal tissues. After resection, a small portion was fixed in 10% formalin for pathological diagnosis while the rest was immediately frozen in liquid nitrogen and stored in a −80°C refrigerator. The clinical pathological information of each recruited cases were fully collected. There were 61 male cases and 15 female cases. Their ages ranged from 40 to 76 years (median age, 60 years). TNM staging was guided by the 7th edition of the AJCC Cancer Staging Manual published by the International Union Against Cancer (UICC) and American Joint Committee on Cancer (AJCC), 2010 [35]. Of 76 cases with ESCC, 5 were stage I, 30 were stage II, 40 were stage III, and 1 were stage IV (Table 1). There were 46 cases with upper and middle esophagus lesions, 30 cases with lower lesions. Meanwhile, 60 cases were classified as well/moderately differentiated and 16 cases as poorly differentiated type. 15 cases of esophageal epithelium atypical hyperplasia, 10 cases of esophagitis and 5 cases of esophageal varices, were obtained from endoscopic biopsy. There were 24 male cases and 6 female cases, aging from 43 to 65 years (median age, 53 years). The Institutional Review Board on Medical Ethics, Zhejiang Province Cancer Hospital approved the method of tissue collection including informed consent.

### Immunohistochemical analysis and pathological evaluation

Immunohistochemistry (IHC) was carried out on tissue sections using the avidin-biotin method and a commercially available kit (Vectastain Elite ABC kit, Vector Laboratories, Burlingame, CA, USA). The reaction was visualized by 3, 30- diaminobenzidine tetrahydrochloride. The nuclei were counterstained with hematoxylin. Negative controls were composed of identically treated histologic sections with the omission of primary antibodies. For IHC studies, the following antibodies were used: HIC1 (Abcam, 330 Cambridge Science Park, Cambridge, CB4 0FL, UK), EphA2 polyclonal antibody (sc-924, dilution 1:200, Santa Cruz, CA, USA), Snail (Abgent Inc, San Diego, California, CA), E-cadherin, Vimentin, Twist, and Zeb1 (Cell Signaling Technology, Danvers, MA, USA). Consecutive sections (4 μm) of paraffin-embedded tissue blocks were prepared and processed for IHC analysis. A semi-quantitative scoring system was used as described previously [29, 30]. In brief, IHC score was determined combining staining frequency and intensity. In detail, the staining frequency score was defined as no staining scored as 0, 1~10% of cells stained as 1, 11~50% of cells stained as 2, 51~80% of cell stained as 3, and 81~100% of cell stained as 4. Staining intensity score was rated on a scale of 0 to 3, with 0 for negative; 1 for weak; 2 for moderate; and 3 for strong staining. Theoretically, the scores could range from 0 to 12. An IHC score of 9~12 was considered as strong immunoreactivity, 5~8 as moderate, 1~4 as considered weak, and 0 as negative. Sections in which the staining could not be well characterized were considered equivocal. Staining was scored independently by two pathologists who were blinded to each other's findings. All conflicting calls on scoring were adjudicated by a third individual.

### RNA extraction and real-time RT-PCR analysis

The mRNA expression level of HIC1 was analyzed by real-time RT-PCR. Total cellular RNAs were extracted using the Trizol (Gibco BRL Life technologies Inc., Rockville, MD, USA) one-step method. A total of 3 μg total RNA was subjected to reverse transcription using M-MLV reverse transcriptase (Promega, San Luis Obispo, CA). The glyceraldehyde phosphate dehydrogenase (GAPDH, TaKaRa Bio, Otsu, Japan) was selected as the internal reference. The primers used in PCR are listed as follows: HIC1 sense 5′-cga cga cta caa gag cag cag c-3′, and antisense 5′-cag gtt gtc acc gaa gct ctc-3′. EphA2 sense 5′-tgt gcc agg cag gct acg-3′, and antisense 5′-ctc caa gca ggg gct ctc a-3′. GAPDH sense 5′-ctg ggc tac act gag cac c-3′, and 5′-aag tgg tcg ttg agg gca atg-3′. The 2-^ΔΔCt^ method was used to calculate relative changes in gene expression determined from real-time RT-PCR experiments. Increased and reduced expressions were defined as the median value of relative gene expression level > 2.0 and < 0.5, respectively.

### DNA extraction, bisulfite modification and real-time methylation-specific PCR

Serial 5-μm-thick sections that contained carcinoma and non-neoplastic tissues were mounted on non-coated glass slides and dried at 37°C overnight. After deparaffinization and staining with Hematoxylin and Eosin (HE), we collected 5000 nuclei from 5 to 10 serial sections using a 27G needle. The collected target cells were treated with 40 μl of 200 μg/ml proteinase K (Sigma-Aldrich) at 42°C, for 72 hours. DNAs were modified by sodium bisulfite using the EpiTect Bisulfite kit (Qiagen Inc.) following manufactory's instructions. Modified DNAs were analyzed by real-time methylation-specific PCR (MSP) on a ABI7500 PCR (ABI Co.) using the SYBR Premix Taq ExTaq Kit (TaKaRa Co. Ltd). The specific primers for detection of HIC1 CpG island methylation and unmethylation were designed according to previous report [36]. The percentage of methylated DNAs in the samples were calculated according to the Ct value and a standard curve, and methylated DNA was scored as previous described [37, 38]. All samples were analyzed with primer sets for both methylated and unmethylated DNA. The relative amount of methylation in each unknown sample was calculated as the percentage methylation = 100 × (number of copies of methylated DNA / [number of copies of methylated + unmethylated DNA]) [46]. The sum of unmethylated plus methylated DNA (U + M) was used as an approximation of the total number of target gene copies. Methylated DNA was scored according to the methylated percentage (0, < 20%; 1, 20%–40%; 2, 40%–60%; 3, 60%–80%; and 4, > 80%; scores of 0, 1–3, and 4 were considered unmethylated, partially methylated, and fully methylated, respectively) [29, 37–39]. The cut off threshold for DNA hypermethylation was set as 20% based on control normal samples and internal quality controls provided in the real-time MSP analysis.

### Plasmids, cell culture, MTT assay, colony formation, migration assay, and immunoblotting

For restoring expression of HIC1 in KYSE410 cell lines, human full-length HIC1 cDNA was inserted into lentivirus vector pHR-SIN-CSIGW under the control of SFFV promoter for stable expression. EphA2 coding sequence and EphA2 shRNA using a recombinant adenovirus gene delivery system were constructed. A blank vector adenovirus gene delivery system was used as a negative control. These products were produced by GeneChem Biomedical Co. Ltd (Shanghai, China). KYSE410 cells were maintained in a 1:1 mixture of RPMI 1640 (Invitrogen, Carlsbad, CA) and Ham's F12 (Nissui Pharmaceutical, Tokyo, Japan) containing 10% fetal bovine serum (FBS, Gibco BRL Life technologies Inc., Rockville, MD, USA) in humidified 5% CO_2_-air at 37°C. MTT assay was performed as previously described [40]. For colony formation assay, cells were plated into a 6-well with 400 cells per well. Cells were then cultured for 5 days and stained with crystal violet. The colonies were counted with more than 50 cells. For migration assays, 5 × 10^4^ cells were seeded into chambers that containing 8 μm pores (Corning, NY, USA) per chamber. After 24 h, the cells that migrated through the membrane into the bottom chamber were fixed and stained by crystal violet. The numbers of migrating cells were counted by randomly selecting nine fields of view per cell line. The protocol for immunoblotting has been described previously [40].

### Statistical analysis

SPSS 17.0 statistical software was adopted for data analysis. Counting data comparisons between groups were subjected to the *χ* ^2^ test and Fisher's exact test. Survival analysis was computed by means of the Kaplan-Meier method and significant levels were assessed by means of the log-rank test. A univariate analysis with the Cox regression model was used to determine prognostic factors, and multivariate analysis with the Cox regression model was used to explore combined effects.
